# Anxiety and Arterial Stiffness in High‐Risk Pregnancies: A Secondary Analysis of a Prospective Cohort Study

**DOI:** 10.1111/1471-0528.18325

**Published:** 2025-08-08

**Authors:** Mekayla Forrest, Maria Matossian, Helena Papacostas Quintanilla, Isabelle Malhamé, Tina Montreuil, Stella S. Daskalopoulou

**Affiliations:** ^1^ Vascular Health Unit Research Institute of McGill University Health Centre Montreal Quebec Canada; ^2^ Department of Medicine McGill University Montreal Quebec Canada; ^3^ Division of General Internal Medicine, Department of Medicine McGill University Health Centre Montreal Quebec Canada; ^4^ Centre for Outcomes Research and Evaluation Research Institute of the McGill University Health Centre Montreal Quebec Canada; ^5^ Department of Educational and Counselling Psychology, Faculty of Education McGill University Montreal Quebec Canada; ^6^ Psychiatry Department Research Institute of McGill University Health Centre Montreal Quebec Canada

**Keywords:** arterial stiffness, Beck Anxiety Inventory, carotid‐femoral pulse wave velocity, high‐risk pregnancy, mood disorders, preeclampsia anxiety

## Abstract

**Objectives:**

In a high‐risk pregnant population with singleton pregnancies, the primary objective was to evaluate the association between anxiety and arterial stiffness (AS) and the secondary objective was to investigate whether anxiety is associated with the incidence of preeclampsia.

**Design:**

Secondary analysis of a prospective cohort study (2012–2016).

**Setting:**

Two tertiary care antenatal clinics in Montreal, Canada.

**Participants:**

High‐risk pregnant individuals with pre‐existing hypertension, diabetes, renal dysfunction, previous preeclampsia, or age ≥ 35 years were included. Exclusion criteria were excessive alcohol or drug use and cardiovascular disease. People with pregnancy loss, incomplete questionnaires and loss to follow‐up were excluded from analysis.

**Methods:**

Participants were enrolled before 14‐weeks' gestation, with follow‐up assessments of AS every 4 weeks until delivery. Anxiety symptoms were assessed every trimester by the Beck Anxiety Inventory and by self‐reported history of emotional disorders (anxiety/depression).

**Main Outcome Measures:**

AS and wave reflection parameters, primarily carotid‐femoral pulse wave velocity and preeclampsia diagnosis.

**Results:**

Of 235 individuals recruited, 161 were included in this secondary analysis. Baseline anxiety symptoms were present in 35.4% of participants. Anxiety was associated with a significant increase in carotid‐femoral pulse wave velocity across gestation, which persisted after adjustments for relevant confounders, in a combined mixed‐effects model (*B* = 0.27, 95% confidence interval [CI] = 0.008–0.530, *p* = 0.04). A severity‐response relationship was observed, where greater anxiety severity correlated with higher AS. Twelve participants (7.5%) developed preeclampsia. The association between anxiety and preeclampsia risk showed a non‐significant trend (odds ratio [OR] = 2.77, 95% CI = 0.84–9.18). However, a history of emotional disorders significantly elevated preeclampsia risk (OR = 3.91, 95% CI = 1.14–13.40), independent of other risk factors.

**Conclusions:**

Anxiety in high‐risk pregnancies is associated with increased AS in a severity‐response manner, and may be associated with preeclampsia risk. Integrating psychological health assessments with traditional obstetric evaluations could enhance the prediction and management of maternal complications.

## 1 Introduction

Pregnancy induces a series of physiological changes. For some, they result in psychological stressors, which may manifest as a fear of pregnancy complications or childbirth, or as anxiety relating to the transition to parenthood [1, 2, 3]. Anxiety is the most prevalent psychiatric disorder and is defined as a strong affective and behavioural response towards a real or imagined future threat or danger [4, 5]. Female sex is associated with a greater risk of developing anxiety‐related disorders [6]. Particularly in pregnancy, the prevalence of anxiety increased by 75% from 2004 to 2014 [7]. It affects approximately 21% of females during pregnancy, becoming more prevalent as the pregnancy progresses [8, 9]. Higher anxiety is linked to various pregnancy complications [10, 11], including hypertensive disorders [7, 12, 13, 14].

Many pregnancies are at high risk of developing complications, such as preeclampsia. The perceived risk of these adverse outcomes may contribute to stress during pregnancy [1, 15]. Preeclampsia is a pregnancy complication characterised by high blood pressure and organ damage, and is a leading cause of maternal and neonatal morbidity and mortality worldwide [16, 17]. While diagnosis occurs in the second half of pregnancy, its pathophysiology begins early, which has led to the development of various prediction models. However, current models, combining clinical characteristics, blood biomarkers, blood pressure measurements and uterine artery Doppler ultrasound, have low to moderate predictive ability for all subtypes of preeclampsia combined [18, 19]. This highlights the need to improve the accuracy of risk assessment models for preeclampsia, particularly for individual risk estimates [20, 21].

Arterial stiffness (AS), a well‐established marker of cardiovascular health, has emerged as a promising predictor of preeclampsia [22, 23, 24, 25, 26]. AS refers to increased stiffness of the arterial wall, or a reduction in the cushioning function of the aorta, which helps limit pulsatility and protect the microvasculature [27, 28, 29, 30]. In pregnancy, increased AS may impair this protective effect and increase cardiovascular strain [31]. AS is evaluated directly through carotid‐femoral pulse wave velocity (cfPWV), the gold standard measure for assessing central AS, with higher values indicating greater AS [27, 28]. Indirectly, AS can be evaluated through pulse wave analysis, which estimates central haemodynamics and wave reflections from waveforms obtained at peripheral sites [28, 29, 30]. Key parameters derived from pulse wave analysis include (a) augmentation pressure, which represents the additional pressure contributed by the reflected wave returning from peripheral sites during late systole, and it adds to the pressure generated by the heart, amplifying the systolic peak and (b) the augmentation index, expressed as the ratio of augmentation pressure to pulse pressure (difference between systolic and diastolic blood pressure). A higher augmentation index means that wave reflections are returning earlier in the cardiac cycle, increasing central blood pressure. This is often a sign of stiffer arteries, which can increase the workload on the heart. Because augmentation index is influenced by heart rate, it is often adjusted to a heart rate of 75 beats per minute (AIx75) [28, 30]. Other parameters include time to wave reflection (T1R), which measures the timing of the wave reflection, whereby lower values indicate higher AS; pulse pressure amplification, defined as the ratio of peripheral to central pulse pressures, whereby lower values indicate stiffer arteries; and the subendocardial viability ratio, an indicator of myocardial oxygen supply and demand, whereby lower values indicate worse myocardial oxygenation [27, 28, 29, 30]. Collectively, these AS parameters provide complementary insights into vascular load, cardiac function and downstream microvascular stress [27, 28, 29, 30]. Accordingly, AS is considered an independent marker of vascular health and has long been included in hypertension guidelines for cardiovascular disease stratification in the general population [32, 33, 34].

In non‐pregnant populations, anxiety is associated with increased AS, hypertension and other cardiovascular outcomes [35, 36, 37, 38]. One study also reported an intensity‐response relationship between higher anxiety severity and increased pulse wave analysis parameters [38]. However, existing evidence in pregnancy is limited. Additional studies are necessary to assess the impact of anxiety on hemodynamic parameters, particularly anxiety experienced at the beginning of pregnancy. In our previous primary analysis of a high‐risk pregnant population, we demonstrated that AS can predict preeclampsia earlier and with greater ability than existing predictive tools, and that altered vascular adaptations were detected using measures of arterial stiffness and wave reflection in the early second trimester of pregnant women who developed preeclampsia compared to those who did not [23, 39]. In the present secondary analysis of this cohort, our primary aim is to evaluate the association between anxiety symptoms and AS, including wave reflection and hemodynamic parameters across pregnancy. We hypothesise that anxiety is associated with increased AS during pregnancy. In an additional exploratory analysis, we aim to investigate the relationship between anxiety and preeclampsia.

## 2 Methods

### 2.1 Study Design and Population

This is a secondary analysis of data collected from our previously reported REVEAL Study (pRedictivE Value of artErial stiffness in pre‐eclAmpsia deveLopment) [23, 39, 40, 41], a prospective cohort study that included high‐risk pregnant individuals with singleton pregnancies. High‐risk criteria for preeclampsia included pre‐existing hypertension, diabetes or renal dysfunction, preeclampsia in a previous pregnancy or a close family member, overweight/obesity, and/or age ≥ 35 years. Exclusion criteria were excessive alcohol use, illicit drug use, history of heart disease, stroke, arrhythmia, or peripheral arterial disease. Ethics approval was granted by McGill University's Institutional Review Board (A04‐M38‐13A). All participants were recruited before 14 weeks gestation and provided written informed consent.

### 2.2 Medical Data Collection

Data were collected through electronic medical records and REVEAL baseline and follow‐up questionnaires (reproductive history, sociodemographic information, family history, medical history, medications, lifestyle habits). Socioeconomic status was calculated by relating participant postal codes to the National Institute of Public Health of Quebec deprivation index database, using 2016 Canadian Census data [42].

### 2.3 Anxiety Symptoms Screening

Self‐reported anxiety symptoms were collected through the 21‐question Beck Anxiety Inventory [43]. Anxiety level was assessed based on the summed score (range 0–63) and grouped according to standardised cutoffs. Anxiety symptoms were classified as either (1) none or sub‐clinical (collectively termed sub‐clinical) (0–7) or clinical (8–63), or (2) ‘minimal’ (0–7), ‘mild’ (8–15), ‘moderate’ (16–25), or ‘severe’ (25–63) [43] (Table S1). Initial assessment was performed in the first trimester (baseline anxiety cohort). Participants who also completed an assessment at Weeks 22–25 were included in a sub‐analysis (follow‐up anxiety cohort) (Figure S1). We also compiled assessments from both timepoints to create the variables ‘persistent anxiety symptoms’ and ‘anxiety at either time point’. History of emotional disorders (anxiety, depression, or other) was also collected through personal medical history questionnaires.

### 2.4 Arterial Stiffness Assessments

AS parameters were collected approximately every 4 weeks starting from 10 to 13 weeks until delivery (Figure S1). AS was assessed non‐invasively by applanation tonometry (SphygmoCor, AtCorMedical, Australia). CfPWV was used to assess central AS. Pulse wave analysis was then performed to collect parameters related to central haemodynamics and wave reflection, including AIx75, T1R, subendocardial viability ratio, peripheral and central blood pressures, mean arterial pressure and pulse pressure amplification. All measurements were taken in duplicate and averaged, with a third taken if the difference exceeded 0.5 m/s for cfPWV or 4% for AIx75.

### 2.5 Preeclampsia Diagnosis

Preeclampsia diagnoses were made by physicians blinded to the study and obtained through medical chart review. Diagnostic criteria for preeclampsia were based on the Society of Obstetrics and Gynaecology Canada guidelines in effect during the study period, including new‐onset or worsening hypertension (systolic blood pressure ≥ 140 mmHg and/or diastolic blood pressure ≥ 90 mmHg), in combination with proteinuria and/or adverse conditions after 20 gestational weeks [44].

### 2.6 Statistical Analysis

Baseline characteristics of participants with and without clinical anxiety symptoms were compared using chi‐square tests and student's t‐tests, and significant differences were identified as confounders. In our primary analysis, we applied linear regression models, both unadjusted and adjusted for baseline characteristics including age, body mass index (BMI), socioeconomic status and relevant confounders: chronic hypertension, family history of preeclampsia and history of emotional disorders. Unadjusted and adjusted mixed‐effects models were further applied to account for repeated measurements across gestation, with random intercepts for each participant with unstructured covariance. We report unstandardised coefficients (*B*) in both models for better interpretability, given the exposure is binary [45] and 95% confidence intervals (CI). In sensitivity analysis, we excluded individuals with relevant confounders. Secondary analyses explored the incremental effect of the Beck Anxiety Inventory standardised cutoffs (minimal, mild, moderate, severe anxiety) on cfPWV. The association between follow‐up or persistent anxiety symptoms and cfPWV was also evaluated. Finally, we performed univariate logistic regression to calculate the odds ratio (OR) of anxiety for the development of preeclampsia, unadjusted and adjusted for baseline characteristics and confounders. In this exploratory analysis, a history of emotional disorders was considered an exploratory variable rather than a confounder. A *p*‐value or adjusted *p*‐value of < 0.05 was considered significant, applying false discovery rate corrections for multiple testing (Benjamini‐Hochberg and Benjamini‐Yekutieli) in the primary analysis [46, 47]. All analyses were performed using STATA/SE version 18 (Stata Corp LP, College Station, USA).

## 3 Results

### 3.1 Participant Baseline Characteristics

**FIGURE 1:**
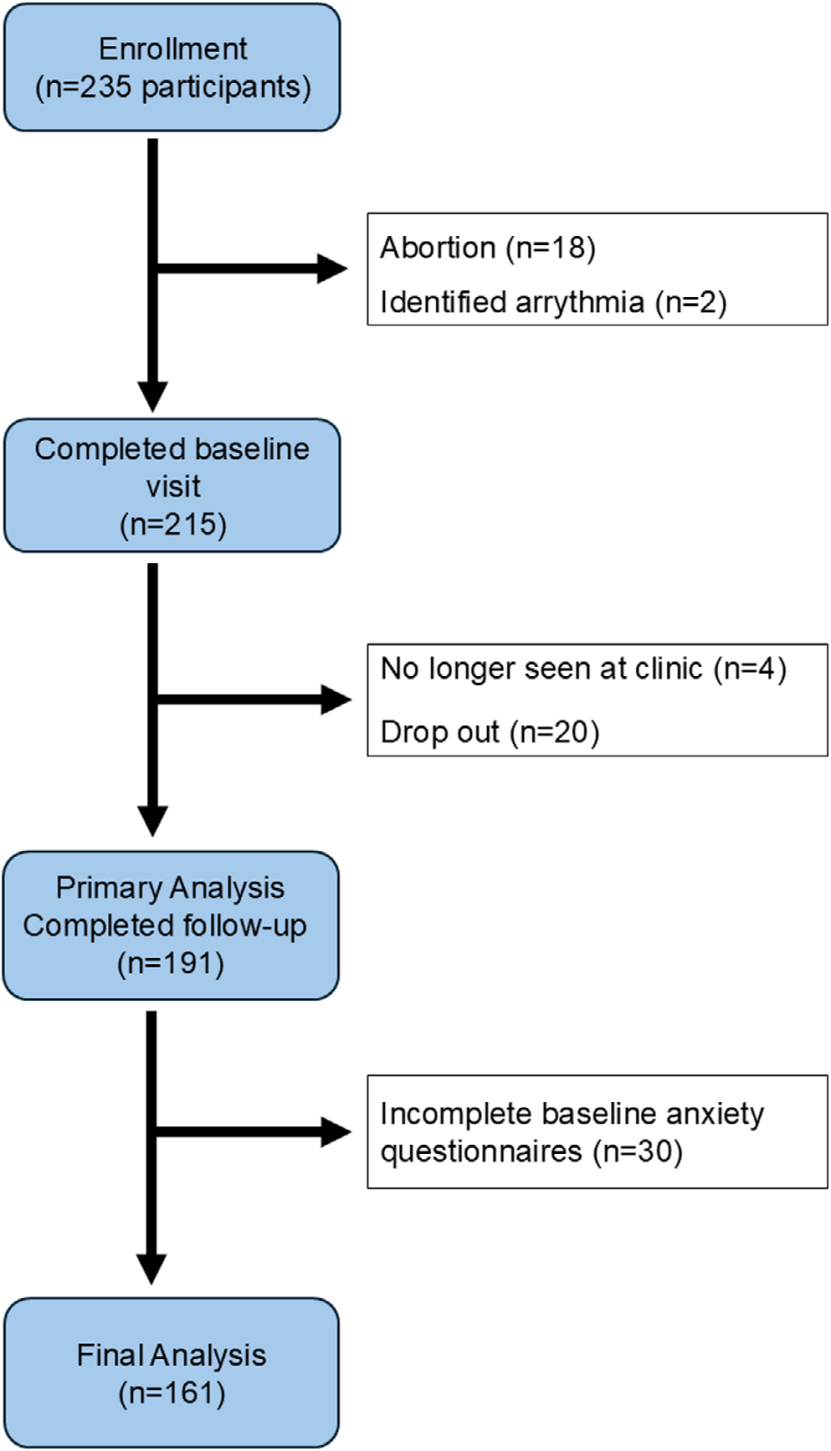
Flowchart of participant enrollment, follow‐up and analysis.

The REVEAL study recruited 235 participants, among whom 191 completed follow‐up visits. Reasons for study drop‐out included pregnancy loss, arrhythmia and loss to follow‐up (Figure 1). Of the enrolled participants, 161 (84.3%) completed the baseline anxiety questionnaire and were included in the ‘baseline anxiety cohort’ (Table S2). Baseline characteristics of participants in the ‘baseline anxiety cohort’ are depicted in Table 1. Compared to participants with sub‐clinical anxiety symptoms, participants with anxiety symptoms more commonly had a family history of preeclampsia (4.8% vs. 14.0%, *p* = 0.04), chronic hypertension (3.9% vs. 22.8%, *p* < 0.001) and history of emotional disorders (12.5% vs. 26.3%, *p* = 0.03). No other differences were observed. Non‐respondents had significantly higher BMI and a larger proportion identified as Black (Table S2). From the baseline anxiety cohort, 140 participants (87.0%) completed the follow‐up anxiety questionnaire between Weeks 22 and 25 (follow‐up anxiety cohort). There were no significant differences in baseline characteristics between follow‐up respondents and non‐respondents, except for pre‐existing chronic hypertension, which was more prevalent in non‐respondents to the follow‐up anxiety questionnaire (Table S3). The incidence of anxiety symptoms in the baseline and follow‐up cohorts was 35.4% and 39.3%, respectively (Table S1).

**TABLE 1:** Comparison of participant baseline characteristics by the presence or absence of anxiety symptoms at baseline.

Baseline characteristics	Overall (*N* = 161)	Sub‐clinical anxiety symptoms (*N* = 104)	Anxiety symptoms (*N* = 57)	*p*
Maternal age (years)	36.7 ± 3.96	36.1 ± 4.76	37.0 ± 3.42	0.17
Pre‐pregnancy body mass index	26.2 ± 6.81	25.5 ± 6.22	27.6 ± 7.63	0.06
Race				
Caucasian	22 (13.7)	17 (16.4)	5 (8.7)	0.18
Black	18 (11.2)	10 (9.6)	8 (14.0)	0.40
Asian	19 (11.8)	13 (12.5)	6 (10.5)	0.71
Hispanic	15 (9.3)	9 (8.7)	6 (10.5)	0.70
Other	87 (54.0)	55 (52.9)	32 (56.1)	0.69
Nulliparous	80 (49.7)	49 (47.1)	31 (54.4)	0.38
Previous pregnancy with gestational hypertension	1 (0.6)	1 (1.0)	0 (0.0)	0.46
Previous pregnancy with preeclampsia	6 (3.7)	2 (1.9)	4 (7.0)	0.10
Previous pregnancy with gestational diabetes	9 (5.6)	8 (7.7)	1 (1.8)	0.12
Previous pregnancy with other complications^a^	14 (8.7)	6 (5.8)	8 (14.0)	0.08
Family history of preeclampsia	13 (8.1)	5 (4.8)	8 (14.0)	0.04*
Preexisting chronic hypertension	17 (10.6)	4 (3.9)	13 (22.8)	< 0.001*
Preexisting diabetes	15 (9.3)	10 (9.6)	5 (8.8)	0.86
Preexisting renal or autoimmune disorder	2 (1.2)	1 (0.1)	1 (1.8)	0.66
Self‐reported history of emotional disorders	28 (17.4)	13 (12.5)	15 (26.3)	0.03*
Indices of socioeconomic status^b^				
Social deprivation factor	2.98 ± 1.33	3.09 ± 1.29	2.77 ± 1.38	0.13
Material deprivation factor	3.34 ± 1.44	3.31 ± 1.45	3.37 ± 1.43	0.79
Combined deprivation factor	3.27 ± 1.39	3.33 ± 1.46	3.18 ± 1.24	0.43

*Note:* Data are presented as mean ± standard deviation or number (percentage).

^a^ Other pregnancy complications include stillbirth, preterm delivery and small for gestational age.

^b^ Socioeconomic status deprivation factors were ranked from 1 to 5 with lower scores indicating greater deprivation.

* Significant at *p*‐value < 0.05.

### 3.2 Association Between Anxiety and Arterial Stiffness Parameters

**FIGURE 2:**
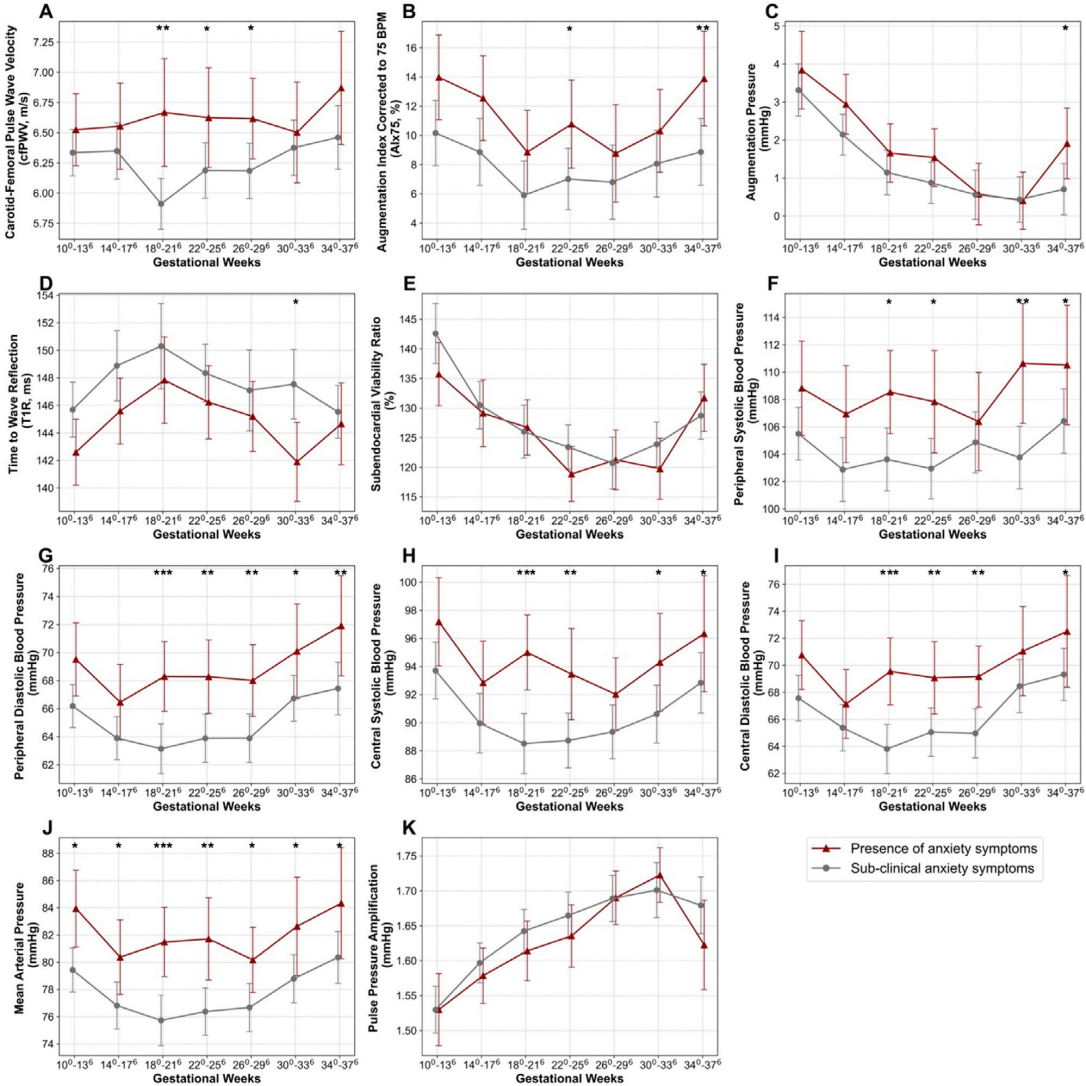
Longitudinal trends of mean arterial stiffness, wave reflection and blood pressure parameters in pregnant individuals, according to the presence of baseline anxiety symptoms. From panel (A–K) Carotid femoral pulse wave velocity (m/s), augmentation index standardised to heart rate (%), augmentation pressure (mmHg), time to wave reflection (ms), subendocardial viability ratio (%), mean peripheral systolic blood pressure (mmHg), peripheral diastolic blood pressure (mmHg), central systolic blood pressure (mmHg), central diastolic blood pressure (mmHg), mean arterial pressure (mmHg) and pulse pressure amplification (PPA), in participants with sub‐clinical baseline anxiety symptoms (grey circles) and with the presence of baseline anxiety symptoms (red triangles). Error bars represent the 95% confidence interval. *p‐adjusted < 0.05, **p‐adjusted < 0.01, ***p‐adjusted < 0.001 (Benjamini‐Yekutieli false discovery correction for multiple comparisons), linear regression, unadjusted for confounders.

AS, wave reflection and blood pressure parameters (Figure 2, Table S4) were compared between participants with baseline anxiety and sub‐clinical anxiety symptoms. Assessments from Weeks 38 to 42 were excluded from analysis due to the high number of deliveries during this period. Linear regression demonstrated that participants with anxiety had significantly higher cfPWV in the second and early third trimesters compared to those with sub‐clinical symptoms (*B* = 0.757, 95% CI = 0.316–1.197; *B* = 0.440, 95% CI = 0.001–0.878; *B* = 0.432, 95% CI = 0.033–0.831; for Weeks 18, 22, 26, respectively) (Figure 2A, Table S4). These differences remained significant at Weeks 18 and 26 after individually adjusting for age, BMI and socioeconomic status, as well as for additional confounders including chronic hypertension, family history of preeclampsia and history of emotional disorders. AIx75 followed a similar trend, with higher values in the anxiety group (Figure 2B). This difference was significant at Weeks 22 and 34 but became non‐significant after adjusting for confounders. T1R was significantly different at Week 30 (*B* = −5.650, 95% CI = −9.707 to −1.583), with and without adjustment for confounders, where the T1R values diverged, decreasing in the anxiety group and increasing in the sub‐clinical anxiety group. Notably, lower T1R indicates greater stiffness. Augmentation pressure and the subendocardial viability ratio were largely similar between groups throughout gestation. Although augmentation pressure was elevated in the anxiety group at Week 34, this difference was not significant after adjusting for confounding variables. Both peripheral and central blood pressures were significantly higher in the anxiety group throughout gestation, while pulse pressure amplification showed no difference (Figure 2).

The associations identified in the linear regression models for individual timepoints were corroborated using mixed‐effects models across gestation. In unadjusted models, anxiety was significantly associated with increased cfPWV (*B* = 0.368, 95% CI = 0.090–0.646) and AIx75 (*B* = 3.290, 95% CI = 0.432–6.159) and a non‐significant trend in reduced T1R (*B* = −2.814, 95% CI = −5.500 to −0.128). The relationship between anxiety and cfPWV persisted after adjustment for age, BMI and confounders including chronic hypertension, family history of preeclampsia and history of emotional disorders, both individually and when combined (*B* = 0.268, 95% CI = 0.008–0.530). Sensitivity analyses excluding participants with these same confounders, advanced maternal age (≥ 35 years), or high BMI (≥ 30 kg/m^2^) did not affect the observed associations. For AIx75, the association remained significant after adjusting for all individual confounders, except for chronic hypertension.

**FIGURE 3:**
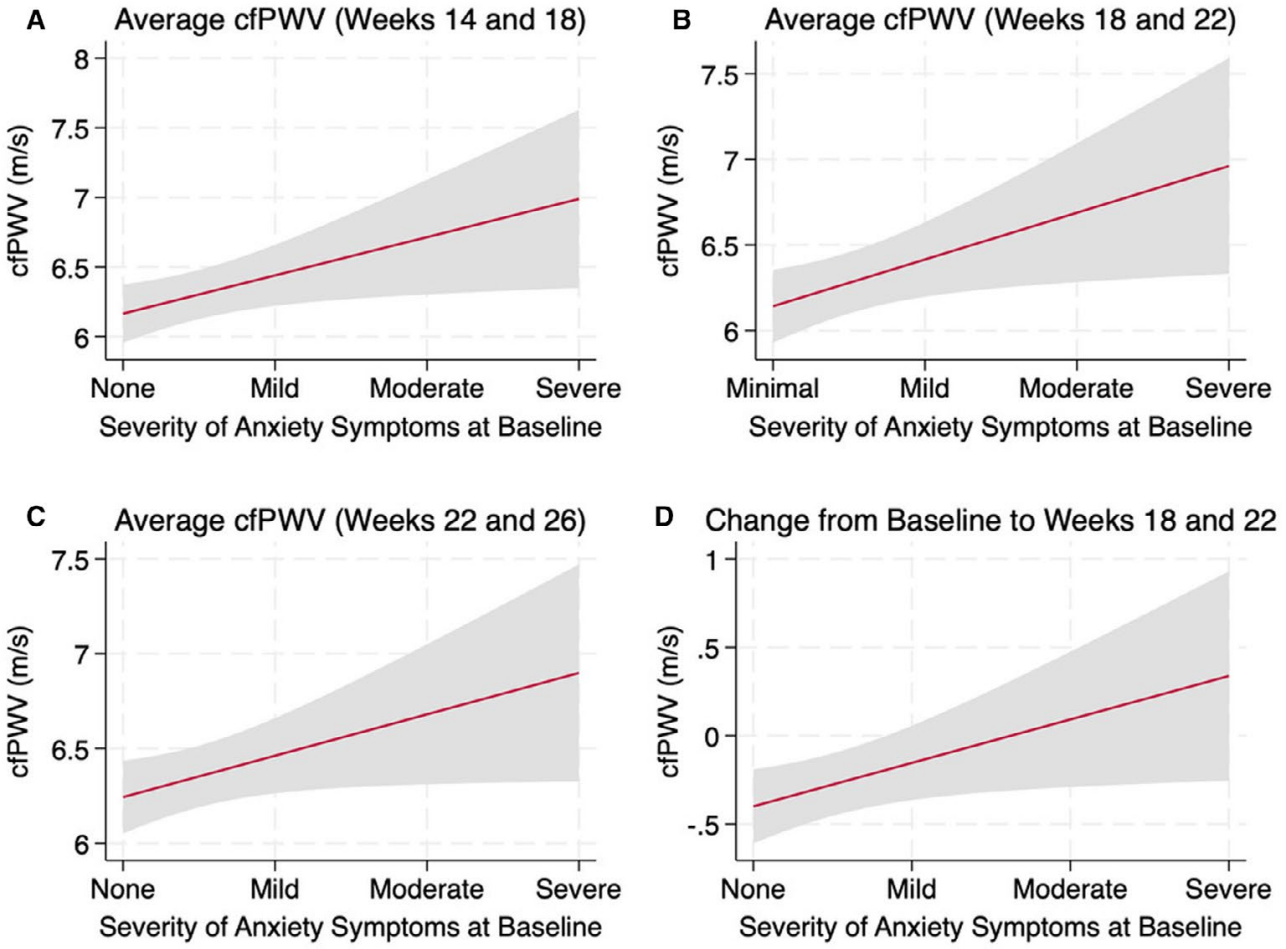
Linear regression of the association between the severity of baseline anxiety symptoms and carotid femoral pulse wave velocity (cfPWV). Baseline Beck Anxiety Inventory rating (minimal, mild, moderate and severe) is positively associated with (A) Average cfPWV at Weeks 14 and 18 (*B* = 0.275, 95% CI 0.032–0.517, *p* = 0.03), (B) Average cfPWV at Weeks 18 and 22 (*B* = 0.273, 95% CI 0.033–0.513, *p* = 0.03), (C) Average cfPWV at Weeks 22 and 26 (*B* = 0.218, 95% CI 0.001–0.435, *p* = 0.049), (D) Change in average cfPWV from baseline (Week 10) to Weeks 18 and 22 (*B* = 0.280, 95% CI 0.022–0.538, *p* = 0.03). Linear regression, unadjusted for confounders. Grey shading represents 95% confidence interval (CI), and red line represents the fitted values.

As cfPWV was significantly higher in individuals with anxiety, we performed secondary analyses to evaluate the incremental effect of anxiety severity on cfPWV. To account for missing data at individual timepoints and for clinical relevance, we averaged cfPWV data within ranges of Weeks 14–18, Weeks 18–22 and Weeks 22–26. We also calculated the mean change in cfPWV from baseline (Week 10) to these timepoints. Linear regression revealed that cfPWV significantly increased with the severity of baseline anxiety symptoms in all three timepoints (Figure 3A–3C) and when evaluating changes from baseline to Weeks 18–22 (Figure 3D). Mixed effect models revealed this association was also significant across gestation (*B* = 0.210, 95% CI = 0.028–0.390).

A sub‐analysis evaluated the association between follow‐up and persistent anxiety symptoms and cfPWV (Figure S2). While no significant difference emerged across all four groups, we observed a trend of increased cfPWV following recent anxiety symptoms. Persistent anxiety was linked to increased cfPWV both in early and late pregnancy, while baseline and follow‐up anxiety were associated with higher cfPWV at mid and late pregnancy, respectively.

### 3.3 Preeclampsia Risk

Twelve participants developed preeclampsia, of whom seven reported baseline anxiety symptoms. We observed a non‐significant trend between anxiety symptoms at baseline and at either timepoint and an increased risk of developing preeclampsia (OR = 2.77, 95% CI = 0.84–9.18; OR = 3.04, 95% CI = 0.79–11.68, respectively) (Table S5). Significance levels decreased further following adjustment for confounders. Interestingly, history of emotional disorders was associated with a significant 3.91‐fold increased preeclampsia risk (95% CI = 1.14–13.40). This remained significant following adjustment for all factors, except family history of preeclampsia.

## 4 Discussion

### 4.1 Principal Findings

In this secondary analysis of a prospective high‐risk pregnant cohort, self‐reported anxiety symptoms were associated with increased cfPWV across gestation, with greater anxiety severity corresponding to increased AS. We also observed a trend between anxiety and preeclampsia, wherein a history of emotional disorders was significantly associated with preeclampsia risk, even after adjustment for confounders.

Pregnant individuals with baseline anxiety symptoms exhibited significantly higher central AS at mid‐pregnancy compared to those without anxiety. These findings align with studies linking anxiety to increased AS in the general population [38, 48]. In normal pregnancy, hemodynamic profiles exhibit a U‐shaped curve in cfPWV, wave reflection and blood pressures [31]. Conversely, participants with anxiety demonstrated consistently higher blood pressure throughout gestation, corroborating earlier studies reporting elevated blood pressure in pregnant people experiencing anxiety [12, 13]. Importantly, differences in cfPWV, AIx75 and T1R occurred at key hemodynamic change points. Specifically, cfPWV was higher in the anxiety group from Weeks 18 to 26. In uncomplicated pregnancies, AS reaches a nadir at this timeframe due to decreased vascular resistance and increased vascular compliance to accommodate blood volume expansion and fetal development [31, 49, 50]. However, in the anxiety group, persistently elevated AS may reflect impaired vascular adaptation, potentially driven by increased sympathetic activity [35, 38, 51]. AIx75 was also higher at Weeks 22 and 34, when unadjusted for confounders, suggesting increased wave reflection during periods of significant hormonal changes and vascular remodelling. The lower T1R throughout aligns with continued increases in systemic vascular resistance [31, 49, 50]. Overall, the faster wave reflections suggest altered autonomic regulation and an inability to fully adapt to late pregnancy demands, further emphasising the potential impact of anxiety on maternal vascular health.

Furthermore, greater anxiety severity was related to higher cfPWV. This aligns with studies in the general population showing associations between severe anxiety symptoms, increased AS and higher blood pressure [35, 38]. Follow‐up data showed a trend of increasing cfPWV with persistent or new‐onset anxiety symptoms. Notably, even a single timepoint where the Beck Anxiety Inventory score exceeded the clinical cut‐off was associated with increased cfPWV. This suggests that, as in the general population, even short‐term anxiety may affect AS [38].

While our findings suggest an association between anxiety and AS, the observational design does not allow for causal inference. It is possible that shared underlying factors, such as high BMI, chronic hypertension, or familial risk, may contribute to both conditions. Additionally, anxiety may reflect the patient's heightened awareness or concern about their pregnancy‐related risks rather than being a direct contributor to vascular changes. Although residual confounding cannot be ruled out, the associations between anxiety and AS remained significant after adjustment for known confounders and in sensitivity analyses, supporting the robustness of our findings. Notably, participants with anxiety also exhibited higher mean arterial pressure and overall elevated blood pressures, aligning with prior studies linking anxiety to elevated blood pressure [12, 13]. These findings raise the possibility that anxiety‐related elevations in blood pressure may contribute to increased AS, or that both may result from shared mechanisms such as autonomic dysregulation or vascular dysfunction associated with anxiety. Although blood pressure was an outcome of interest in this study and therefore not included as a covariate, future research should explore potential mediating pathways to clarify directionality and underlying mechanisms.

There is substantial evidence linking anxiety to cardiovascular disease in the general population, yet research is limited in pregnancy [7, 10, 11, 12, 13, 14, 52, 53]. Emerging evidence from our group and others links AS and preeclampsia [22, 23, 24, 25, 26, 39]. Our findings herein suggest that anxiety, an exacerbating but often overlooked factor for AS, may also represent a potential risk factor for preeclampsia. While previous studies in general‐risk pregnancy cohorts found no association between psychological stress and preeclampsia [52, 53], anxiety may be more relevant in high‐risk populations, whereby traditional vascular risk factors are already present. Perinatal anxiety is also associated with fetal developmental issues through alterations in brain structure and function [54, 55], yet remains underdiagnosed and undertreated [56, 57].

### 4.2 Clinical Implications

Overall, our study highlights the need for obstetrical care addressing both physical and psychological health. Although we do not establish causation, this is, to our knowledge, the first study to report associations between anxiety and increased AS in high‐risk pregnancy. This relationship calls for further exploration, with several factors warranting consideration in future research.

Participants with chronic hypertension exhibited higher rates of anxiety symptoms, possibly attributed to awareness of their elevated risk of pregnancy complications. This underscores the need to consider psychological aspects in clinical care [1, 15]. A significant loss of participants with chronic hypertension at follow‐up was also noted; although adjusting for chronic hypertension did not alter significance in our analyses. Additionally, non‐respondents to the baseline anxiety questionnaire shifted the study cohort's demographic characteristics, reducing the representation of Black individuals [58] and those with higher BMI. This loss in study diversity may influence the generalisability of our findings, as race and BMI are important factors in obstetrical risk assessment [59] and potentially anxiety in pregnancy [60]. Future studies should be designed to ensure large, diverse study populations to mitigate these considerations and ensure generalisability of findings.

This study relied on self‐reported questionnaires completed at specific timepoints, but lacks the depth of a diagnostic impression rendered by a clinician. While the Beck Anxiety Inventory is widely used for general anxiety screening, its applicability in pregnant populations is limited as it measures somatic anxiety symptoms, which can overlap with physical pregnancy symptoms [43, 61, 62]. Alternatively, the Generalised Anxiety Disorder 7 assesses psychological symptoms and is validated in diverse populations, including pregnant individuals [63, 64]. While newer questionnaires evaluating anxiety specifically in pregnancy exist [65, 66, 67, 68], they lack universal validation and there is minimal consensus on a reliable gold standard questionnaire in pregnancy [69]. Future research should aim to develop and/or validate anxiety measurement instruments tailored to the unique experiences of pregnant individuals.

### 4.3 Research Implications

Our results underscore the need for large, diverse studies to validate these findings. Addressing factors such as diverse participant representations, precision of anxiety measurements and exploring mechanisms linking anxiety and AS in pregnancy will enhance our ability to identify and mitigate risks associated with anxiety, increased AS and preeclampsia. We are currently in the process of addressing these knowledge gaps in a larger cohort [70].

### 4.4 Strengths and Limitations

Our study's strength lies in its multiple timepoint assessments of AS and anxiety across pregnancy. Importantly, focusing on a high‐risk cohort allowed us to examine the population closely monitored in clinical settings. Although this approach is clinically relevant, it may limit the generalisability to all pregnancies. Several limitations warrant consideration. The small sample size, particularly the number of participants with severe anxiety and preeclampsia, limited our statistical power. Additionally, shifts in study cohort demographics, particularly chronic hypertension, race and BMI, could have introduced potential biases. Lastly, while the Beck Anxiety Inventory provided a snapshot of anxiety symptoms, it may have lacked the diagnostic depth necessary for comprehensive clinical assessment.

## 5 Conclusions

Overall, this work highlights an association between anxiety and AS in pregnancy in a severity‐response manner, and suggests that anxiety may contribute to the vascular profiles observed in individuals with preeclampsia. While further research is required to confirm findings and clarify directionality, our study underscores the need for whole‐person obstetrical care that considers both physical and psychological health. Understanding the interplay between anxiety and AS in pregnancy may support more comprehensive risk assessment and targeted prevention, improving outcomes for both mothers and their infants.

## Author Contributions

S.S.D., M.F., M.M. and H.P.Q. contributed to the conceptualisation of the manuscript and the outline of presented ideas. M.F. performed the statistical analysis, literature review, data collection and drafted the final version of the manuscript. M.M. contributed to the statistical analysis, literature review, data collection and drafted sections of the manuscript. H.P.Q. provided scientific input, guidance and assisted with data collection and analysis. T.M. and I.M. contributed clinical review, scientific input and guidance throughout. S.S.D. provided clinical review, scientific input, guidance, assisted with data analysis and acquired funding. All authors critically reviewed and approved the final version of the manuscript. We extend our heartfelt thanks to the participants, volunteers and hospital staff who generously contributed their time and support to this study.

## Conflicts of Interest

The authors declare no conflicts of interest.

## Supporting information


**Data S1:** bjo18325‐sup‐0001‐Supinfo.docx.

## Data Availability

The data that support the findings of this study are available from the corresponding author upon reasonable request.
