# Supplementary material for: Anxiety and Arterial Stiffness in High‐Risk Pregnancies: A Secondary Analysis of a Prospective Cohort Study
Source: BJOG. 2025 Aug 8;132(12):1833–43. doi: 10.1111/1471-0528.18325 (PMC12501691; doi:10.1111/1471-0528.18325)
Supplement: Supplementary file 1 — Data S1: bjo18325‐sup‐0001‐Supinfo.docx. [file BJO-132-1833-s001.docx]

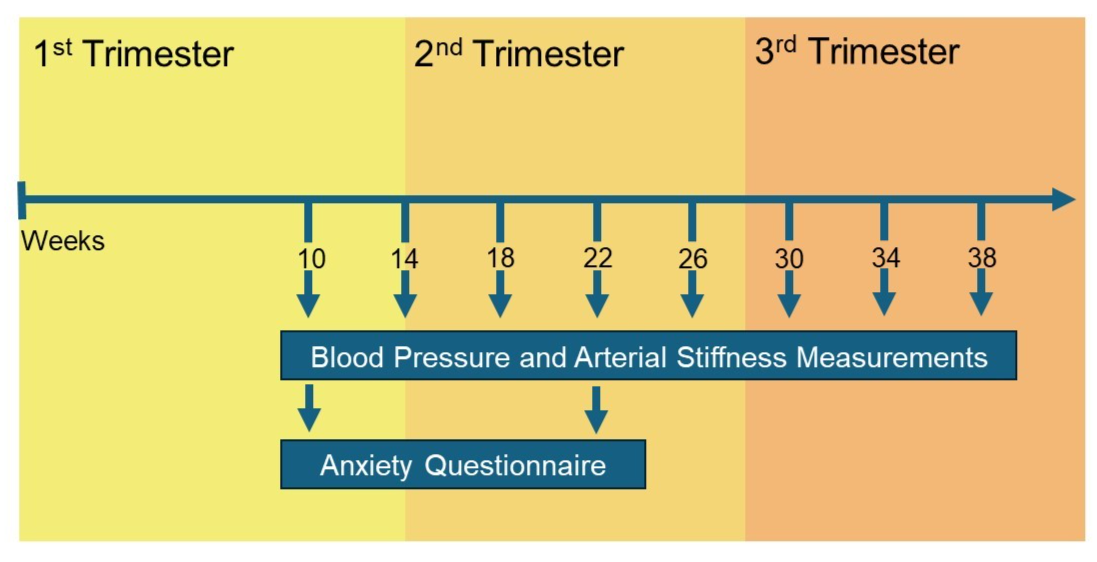


**Supplemental Figure 1:** REVEAL timeline for arterial stiffness assessments and anxiety questionnaires.


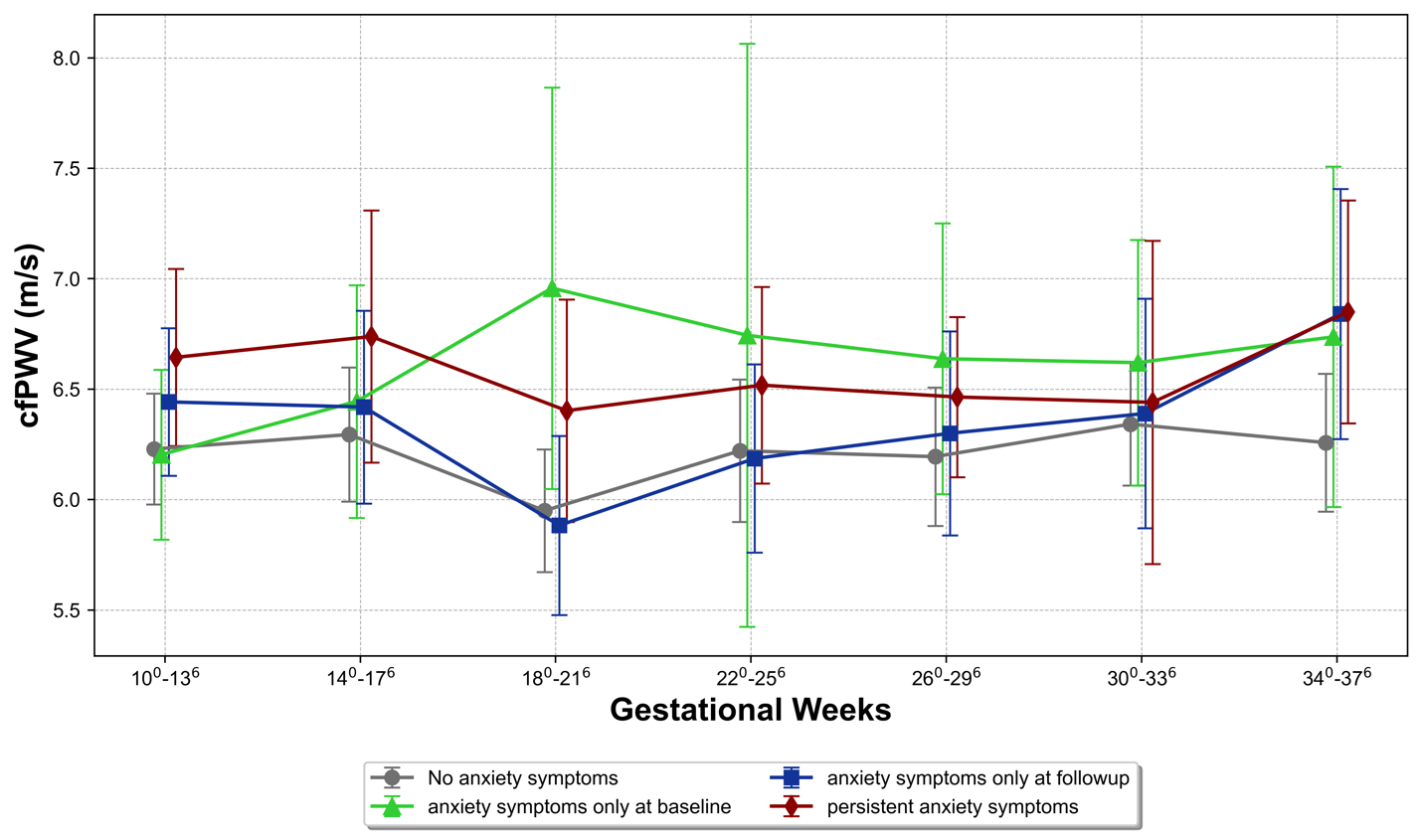


| Week | Sub-clinical anxiety symptoms | Baseline anxiety | p-value | Follow-up anxiety | p-value | Persistent anxiety | p-value |
| --- | --- | --- | --- | --- | --- | --- | --- |
| 10 | 6.22 ± 1.01 | 6.20 ± 0.73 | 0.542 | 6.44 ± 0.83 | 0.161 | 6.64 ± 1.02 | **0.046*** |
| 14 | 6.29 ± 1.18 | 6.44 ± 1.01 | 0.317 | 6.41 ± 1.00 | 0.325 | 6.73 ± 1.24 | 0.095 |
| 18 | 5.95 ± 0.94 | 6.96 ± 1.67 | **0.028*** | 5.88 ± 0.95 | 0.604 | 6.40 ± 1.15 | 0.066 |
| 22 | 6.22 ± 1.09 | 6.75 ± 1.90 | 0.233 | 6.19 ± 0.95 | 0.551 | 6.52 ± 1.13 | 0.145 |
| 26 | 6.19 ± 1.09 | 6.63 ± 1.03 | 0.113 | 6.30 ± 1.05 | 0.356 | 6.46 ± 0.92 | 0.137 |
| 30 | 6.34 ± 0.99 | 6.62 ± 0.90 | 0.198 | 6.39 ± 1.19 | 0.437 | 6.44 ± 1.49 | 0.404 |
| 34 | 6.26 ± 1.11 | 6.74 ± 1.47 | 0.137 | 6.84 ± 1.32 | **0.043*** | 6.85 ± 1.06 | **0.030*** |

**Supplemental Figure 2: Longitudinal trends of mean cfPWV measures in the follow up cohort, according to the presence of anxiety symptoms at two timepoints throughout pregnancy.** Mean carotid femoral pulse wave velocity (cfPWV) in participants with clinical anxiety symptoms at: neither timepoint (grey circles), baseline only (green triangles), follow-up only (blue squares), and both timepoints (red diamonds). Error bars represent the 95% confidence interval. Corresponding table represents the cfPWV (mean ± standard deviation) for each group at each time point, comparing mean cfPWV to the group with subclinical anxiety symptoms (Welch’s t-test). * Significant at p-value <0.05

**Supplemental Table 1: Beck Anxiety Inventory Scoring for anxiety symptoms and participant scores at baseline and follow-up**

| **Baseline Anxiety Cohort** | | | | |
| --- | --- | --- | --- | --- |
| **Total BAI score** | **Standardized clinical cut-off** | **Number of participants (n=161)** | **Grouping for main analysis** | **Number of participants** |
| 0 | Minimal anxiety | 18 (11.2%) | Sub-clinical anxiety symptoms | 104 (64.6%) |
| 1-7 |  | 86 (53.4%) |  |  |
| 8-15 | Mild anxiety | 40 (24.8%) | Presence of anxiety symptoms | 57 (35.4%) |
| 16-25 | Moderate anxiety | 14 (8.7%) |  |  |
| 26-63 | Severe anxiety | 3 (1.9%) |  |  |
| **Follow-up Anxiety Cohort** | | | | |
| **Total BAI score** | **Standardized clinical cut-off** | **Number of participants (n=140)** | **Grouping for main analysis** | **Number of participants** |
| 0 | Minimal anxiety | 14 (10.0%) | Sub-clinical anxiety symptoms | 85 (60.7%) |
| 1-7 |  | 71 (50.7%) |  |  |
| 8-15 | Mild anxiety | 36 (25.7%) | Presence of anxiety symptoms | 55 (39.3%) |
| 16-25 | Moderate anxiety | 13 (9.3%) |  |  |
| 26-63 | Severe anxiety | 6 (4.3%) |  |  |

**Supplemental Table 2: Baseline characteristics of enrolled participants by anxiety questionnaire respondence status**

| Baseline characteristics | Respondent:  included in anxiety cohort (n=161) | Non-respondent: excluded from anxiety cohort (n=30) | P value |
| --- | --- | --- | --- |
| Maternal age | 36.7 ± 3.96 | 37.0 ± 4.81 | 0.70 |
| Pre-pregnancy body mass index | 26.2 ± 6.81 | 29.2 ± 6.68 | **0.03*** |
| **Race**  Caucasian  Black  Asian  Hispanic  Other | 22 (13.7)  18 (11.2)  19 (11.8)  15 (9.3)  87 (54.0) | 5 (16.7)  13 (43.3)  2 (6.7)  2 (2.7)  8 (26.7) | 0.67  **<0.001***  0.41  0.64  **0.006*** |
| Nulliparous | 80 (49.7) | 14 (46.7) | 0.89 |
| Previous pregnancy with gestational hypertension | 1 (0.6) | 0 (0.0) | 0.67 |
| Previous pregnancy with preeclampsia | 6 (3.7) | 0 (0.0) | 0.29 |
| Previous pregnancy with gestational diabetes | 9 (5.6) | 1 (3.3) | 0.63 |
| Previous pregnancy with other complications*^a^* | 14 (8.7) | 2 (6.7) | 0.75 |
| Family history of preeclampsia | 13 (8.1) | 2 (6.7) | 0.83 |
| Preexisting chronic hypertension | 17 (10.6) | 1 (3.3) | 0.21 |
| Preexisting diabetes | 15 (9.3) | 3 (10.0) | 0.91 |
| Preexisting renal or autoimmune disorder | 2 (1.2) | 0 (0.0) | 0.55 |

Data are presented as number (percentage) or mean ± standard deviation. *^a^Other pregnancy complications* include stillbirth, preterm delivery, and small for gestational age. * Significant at p-value <0.05

**Supplemental Table 3: Baseline characteristics of participants by follow-up questionnaire respondence status**

| Baseline characteristics | Completed follow up questionnaire (n=140) | Did not complete follow up questionnaire (n=21) | P value |
| --- | --- | --- | --- |
| Maternal age | 36.5 ± 3.93 | 37.5 ± 4.13 | 0.31 |
| Pre-pregnancy body mass index | 25.8 ± 6.60 | 28.8 ± 7.74 | 0.06 |
| **Race**  Caucasian  Black  Asian  Hispanic  Other | 20 (14.3)  14 (10.0)  16 (11.4)  14 (10.0)  76 (54.3) | 2 (9.5)  4 (19.0)  3 (14.3)  1 (4.8)  11 (52.4) | 0.55  0.22  0.71  0.44  0.87 |
| Nulliparous | 70 (50.0) | 10 (47.6) | 0.84 |
| Previous pregnancy with gestational hypertension | 1 (0.7) | 0 (0.0) | 0.70 |
| Previous pregnancy with preeclampsia | 6 (4.3) | 0 (0.0) | 0.33 |
| Previous pregnancy with gestational diabetes | 9 (6.4) | 0 (0.0) | 0.23 |
| Previous pregnancy with other complications*^a^* | 13 (9.3) | 1 (4.8) | 0.49 |
| Family history of preeclampsia | 13 (9.3) | 0 (0.0) | 0.15 |
| Preexisting chronic hypertension | 11 (7.9) | 6 (28.6) | **0.004*** |
| Preexisting diabetes | 12 (8.6) | 3 (14.3) | 0.40 |
| Preexisting renal or autoimmune disorder | 1 (0.7) | 1 (4.8) | 0.12 |
| Self-reported history of emotional disorders | 25 (17.9) | 3 (14.3) | 0.69 |
| **Indices of socioeconomic status*^b^***  Social deprivation factor  Material deprivation factor  Combined deprivation factor | 3.0 ± 1.31  3.3 ± 1.45  3.3 ± 1.40 | 2.90 ± 1.48  3.6 ± 1.40  3.3 ± 1.32 | 0.66  0.78  0.71 |

Data are presented as number (percentage) or mean ± standard deviation. *^a^Other pregnancy complications* include stillbirth, preterm delivery, and small for gestational age. *^b^Socioeconomic status deprivation factors* were ranked from 1-5 with lower scores indicating greater deprivation. * Significant at p-value <0.05

**Supplemental Table 4: Mean arterial stiffness, wave reflection, and hemodynamic parameters between participants with and without baseline anxiety symptoms**

| Parameter | Sub-clinical anxiety symptoms | Anxiety symptoms | Coefficient (B) | Lower 95% CI | Upper 95% CI | p-adjusted |
| --- | --- | --- | --- | --- | --- | --- |
| **Carotid femoral pulse wave velocity (cfPWV, m/s)** | | | | | | |
| Weeks 10–13.6 | 6.34 ± 0.96 | 6.52 ± 1.07 | 0.190 | -0.157 | 0.536 | 0.15 |
| Weeks 14–17.6 | 6.35 ± 1.10 | 6.55 ± 1.14 | 0.205 | -0.219 | 0.630 | 0.15 |
| Weeks 18–21.6 | 5.91 ± 0.91 | 6.67 ± 1.40 | 0.757 | 0.316 | 1.197 | **0.003*** |
| Weeks 22–25.6 | 6.19 ± 0.99 | 6.63 ± 1.33 | 0.440 | 0.001 | 0.878 | **0.04*** |
| Weeks 26–29.6 | 6.18 ± 1.02 | 6.62 ± 1.10 | 0.432 | 0.033 | 0.831 | **0.05*** |
| Weeks 30–33.6 | 6.38 ± 1.02 | 6.50 ± 1.20 | 0.126 | -0.322 | 0.576 | >0.99 |
| Weeks 34–37.6 | 6.46 ± 1.17 | 6.87 ± 1.42 | 0.410 | -0.098 | 0.917 | 0.23 |
| Across gestation | 6.27 ± 1.03 | 6.62 ± 1.23 | 0.368 | 0.090 | 0.646 | **0.02*** |
| **Augmentation index adjusted to heartrate of 75 beats per minute (AIx75, %)** | | | | | | |
| Weeks 10–13.6 | 10.16 ± 11.14 | 13.98 ± 10.36 | 3.815 | 0.039 | 7.592 | 0.13 |
| Weeks 14–17.6 | 8.86 ± 10.98 | 12.55 ± 9.93 | 3.685 | -0.172 | 7.541 | 0.08 |
| Weeks 18–21.6 | 5.91 ± 9.98 | 8.86 ± 9.65 | 2.950 | -0.806 | 6.706 | 0.11 |
| Weeks 22–25.6 | 7.01 ± 9.49 | 10.77 ± 9.95 | 3.759 | 0.100 | 7.417 | **0.03*** |
| Weeks 26–29.6 | 6.80 ± 11.21 | 8.76 ± 10.76 | 1.966 | -2.322 | 6.254 | 0.20 |
| Weeks 30–33.6 | 8.06 ± 10.17 | 10.31 ± 9.37 | 2.245 | -1.522 | 6.013 | 0.11 |
| Weeks 34–37.6 | 8.87 ± 10.31 | 13.89 ± 10.04 | 5.022 | 0.978 | 9.067 | **0.006*** |
| Across gestation | 8.07 ± 10.55 | 11.33 ± 10.12 | 3.290 | 0.432 | 6.159 | **0.04*** |
| **Augmentation pressure (mmHg)** | | | | | | |
| Weeks 10–13.6 | 3.31 ± 3.44 | 3.84 ± 3.68 | 0.524 | -0.690 | 1.738 | >0.99 |
| Weeks 14–17.6 | 2.14 ± 2.58 | 2.94 ± 2.69 | 0.800 | -0.149 | 1.749 | 0.13 |
| Weeks 18–21.6 | 1.15 ± 2.51 | 1.66 ± 2.63 | 0.512 | -0.455 | 1.479 | 0.27 |
| Weeks 22–25.6 | 0.87 ± 2.47 | 1.54 ± 2.51 | 0.663 | -0.275 | 1.600 | 0.11 |
| Weeks 26–29.6 | 0.56 ± 2.91 | 0.58 ± 2.61 | 0.015 | -1.070 | 1.100 | 0.53 |
| Weeks 30–33.6 | 0.43 ± 2.66 | 0.40 ± 2.49 | -0.031 | -1.021 | 0.959 | 0.43 |
| Weeks 34–37.6 | 0.70 ± 3.08 | 1.91 ± 2.93 | 1.205 | 0.023 | 2.388 | **0.02*** |
| Across gestation | 1.39 ±3.01 | 1.91 ± 3.05 | 0.504 | -0.247 | 1.270 | 0.23 |
| **Time to wave reflection (T1R, ms)** | | | | | | |
| Weeks 10–13.6 | 145.69 ± 10.00 | 142.60 ± 8.64 | -3.089 | -6.385 | 0.207 | 0.23 |
| Weeks 14–17.6 | 148.88 ± 12.06 | 145.59 ± 8.10 | -3.294 | -7.290 | 0.702 | 0.25 |
| Weeks 18–21.6 | 150.30 ± 13.16 | 147.83 ± 10.74 | -2.472 | -7.130 | 2.186 | 0.41 |
| Weeks 22–25.6 | 148.34 ± 9.49 | 146.22 ± 8.80 | -2.114 | -5.616 | 1.387 | 0.41 |
| Weeks 26–29.6 | 147.09 ± 13.16 | 145.19 ± 8.23 | -1.901 | -6.427 | 2.624 | 0.48 |
| Weeks 30–33.6 | 147.54 ± 11.25 | 141.89 ± 9.51 | -5.650 | -9.707 | -1.583 | **0.048*** |
| Weeks 34–37.6 | 145.53 ± 8.72 | 144.66 ± 9.34 | -0.870 | -4.360 | 2.620 | 0.62 |
| Across gestation | 147.54 ± 11.22 | 144.83 ± 9.2 | -2.814 | -5.500 | -0.128 | 0.06 |
| **Subendocardial viability ratio (%)** | | | | | | |
| Weeks 10–13.6 | 142.60 ± 25.37 | 135.72 ± 19.23 | -6.881 | -14.970 | 1.207 | 0.26 |
| Weeks 14–17.6 | 130.49 ± 19.17 | 129.13 ± 19.32 | -1.365 | -8.333 | 5.603 | 0.94 |
| Weeks 18–21.6 | 126.01 ± 19.05 | 126.72 ± 16.02 | 0.708 | -6.080 | 7.497 | 0.75 |
| Weeks 22–25.6 | 123.41 ± 17.01 | 118.87 ± 15.34 | -4.541 | -10.764 | 1.682 | 0.10 |
| Weeks 26–29.6 | 120.71 ± 19.46 | 121.27 ± 16.27 | 0.559 | -6.560 | 7.678 | 0.47 |
| Weeks 30–33.6 | 123.89 ± 16.76 | 119.75 ± 17.02 | -4.141 | -10.557 | 2.276 | 0.09 |
| Weeks 34–37.6 | 128.71 ± 18.31 | 131.72 ± 17.83 | 3.012 | -4.073 | 10.097 | 0.15 |
| Across gestation | 128.53 ± 20.84 | 126.41 ± 18.27 | -2.728 | -7.707 | 2.252 | 0.31 |
| **Peripheral systolic blood pressure** **(mmHg)** | | | | | | |
| Weeks 10–13.6 | 105.49 ± 9.87 | 108.83 ± 13.04 | 3.333 | -0.336 | 7.002 | 0.20 |
| Weeks 14–17.6 | 102.88 ± 11.28 | 106.94 ± 12.14 | 4.063 | -0.117 | 8.243 | 0.08 |
| Weeks 18–21.6 | 103.62 ± 10.30 | 108.54 ± 10.52 | 4.918 | 1.088 | 8.749 | **0.01*** |
| Weeks 22–25.6 | 102.95 ± 10.14 | 107.84 ± 12.52 | 4.889 | 0.775 | 9.004 | **0.01*** |
| Weeks 26–29.6 | 104.87 ± 10.24 | 106.39 ± 12.29 | 1.514 | -2.537 | 5.566 | 0.25 |
| Weeks 30–33.6 | 103.76 ± 10.70 | 110.64 ± 15.01 | 6.886 | 2.365 | 11.406 | **0.001*** |
| Weeks 34–37.6 | 106.42 ± 11.02 | 110.52 ± 14.94 | 4.102 | -0.474 | 8.679 | **0.03*** |
| Across gestation | 104.31 ± 10.53 | 108.54 ± 12.97 | 4.159 | 1.136 | 7.182 | **0.03*** |
| **Peripheral diastolic blood pressure** **(mmHg)** | | | | | | |
| Weeks 10–13.6 | 66.19 ± 7.87 | 69.51 ± 9.86 | 3.324 | 0.469 | 6.178 | 0.06 |
| Weeks 14–17.6 | 63.90 ± 7.43 | 66.46 ± 9.23 | 2.561 | -0.352 | 5.475 | 0.11 |
| Weeks 18–21.6 | 63.14 ± 7.92 | 68.30 ± 8.58 | 5.156 | 2.140 | 8.171 | **<0.001*** |
| Weeks 22–25.6 | 63.89 ± 7.90 | 68.28 ± 8.76 | 4.395 | 1.330 | 7.460 | **0.004*** |
| Weeks 26–29.6 | 63.89 ± 7.96 | 68.01 ± 8.75 | 4.115 | 1.079 | 7.152 | **0.005*** |
| Weeks 30–33.6 | 66.73 ± 7.62 | 70.09 ± 11.54 | 3.361 | 0.009 | 6.713 | **0.02*** |
| Weeks 34–37.6 | 67.44 ± 8.79 | 71.91 ± 12.26 | 4.473 | 0.771 | 8.174 | **0.007*** |
| Across gestation | 65.03 ± 8.04 | 68.96 ± 9.99 | 3.779 | 1.610 | 5.948 | **0.007*** |
| **Central systolic blood pressure** **(mmHg)** | | | | | | |
| Weeks 10–13.6 | 93.71 ± 10.15 | 97.19 ± 11.23 | 3.473 | -0.173 | 7.118 | 0.17 |
| Weeks 14–17.6 | 89.96 ± 10.17 | 92.84 ± 9.90 | 2.888 | -0.818 | 6.594 | 0.17 |
| Weeks 18–21.6 | 88.51 ± 9.051 | 95.00 ± 9.25 | 6.483 | 3.039 | 9.927 | **<0.001*** |
| Weeks 22–25.6 | 88.73 ± 8.68 | 93.46 ± 11.10 | 4.729 | 1.138 | 8.320 | **0.007*** |
| Weeks 26–29.6 | 89.35 ± 8.48 | 92.02 ± 8.58 | 2.673 | -0.577 | 5.923 | 0.06 |
| Weeks 30–33.6 | 90.61 ± 9.16 | 94.30 ± 11.51 | 3.687 | -0.141 | 7.515 | **0.03*** |
| Weeks 34–37.6 | 92.84 ± 9.86 | 96.34 ± 12.85 | 3.496 | -0.790 | 7.782 | **0.04*** |
| Across gestation | 90.68 ± 9.59 | 94.47 ± 10.71 | 3.491 | 0.821 | 6.161 | **0.02*** |
| **Central diastolic blood pressure** **(mmHg)** | | | | | | |
| Weeks 10–13.6 | 67.57 ± 8.43 | 70.75 ± 9.10 | 3.187 | 0.186 | 6.188 | 0.10 |
| Weeks 14–17.6 | 65.38 ± 8.26 | 67.13 ± 8.51 | 1.751 | -1.314 | 4.816 | 0.35 |
| Weeks 18–21.6 | 63.81 ± 7.72 | 69.55 ± 8.58 | 5.744 | 2.700 | 8.788 | **<0.001*** |
| Weeks 22–25.6 | 65.05 ± 7.96 | 69.07 ± 9.16 | 4.022 | 0.885 | 7.159 | **0.008*** |
| Weeks 26–29.6 | 64.97 ± 8.06 | 69.17 ± 7.49 | 4.201 | 1.200 | 7.203 | **0.004*** |
| Weeks 30–33.6 | 68.46 ± 8.74 | 71.05 ± 10.91 | 2.592 | -1.051 | 6.235 | 0.07 |
| Weeks 34–37.6 | 69.33 ± 8.82 | 72.51 ± 12.84 | 3.186 | -0.849 | 7.221 | **0.05*** |
| Across gestation | 66.44 ± 8.47 | 69.85 ± 9.59 | 3.062 | 0.800 | 5.324 | **0.02*** |
| **Mean arterial blood pressure** **(mmHg)** | | | | | | |
| Weeks 10–13.6 | 79.43 ± 8.07 | 83.95 ± 10.17 | 4.518 | 1.471 | 7.565 | **0.01*** |
| Weeks 14–17.6 | 76.82 ± 8.29 | 80.37 ± 9.39 | 3.554 | 0.408 | 6.699 | **0.04*** |
| Weeks 18–21.6 | 75.73 ± 7.92 | 81.48 ± 8.72 | 5.754 | 2.633 | 8.875 | **<0.001*** |
| Weeks 22–25.6 | 76.37 ± 7.90 | 81.71 ± 9.99 | 5.341 | 2.060 | 8.622 | **0.001*** |
| Weeks 26–29.6 | 76.67 ± 7.91 | 80.17 ± 7.75 | 3.496 | 0.462 | 6.530 | **0.01*** |
| Weeks 30–33.6 | 78.78 ± 7.87 | 82.66 ± 11.93 | 3.852 | 0.232 | 7.473 | **0.02*** |
| Weeks 34–37.6 | 80.36 ± 8.68 | 84.33 ± 12.85 | 3.974 | -0.006 | 7.954 | **0.02*** |
| Across gestation | 77.81 ± 8.23 | 82.10 ± 10.21 | 4.034 | 1.662 | 6.406 | **0.005*** |
| **Pulse pressure amplification** | | | | | | |
| Weeks 10–13.6 | 1.53 ± 0.17 | 1.53 ± 0.19 | 0.000 | -0.060 | 0.060 | >0.99 |
| Weeks 14–17.6 | 1.60 ± 0.14 | 1.58 ± 0.14 | -0.018 | -0.068 | 0.031 | 0.63 |
| Weeks 18–21.6 | 1.64 ± 0.13 | 1.61 ± 0.15 | -0.028 | -0.080 | 0.024 | 0.26 |
| Weeks 22–25.6 | 1.66 ± 0.15 | 1.64 ± 0.15 | -0.029 | -0.086 | 0.028 | 0.21 |
| Weeks 26–29.6 | 1.69 ± 0.15 | 1.69 ± 0.12 | 0.001 | -0.053 | 0.055 | 0.52 |
| Weeks 30–33.6 | 1.70 ± 0.18 | 1.72 ± 0.13 | 0.022 | -0.040 | 0.083 | 0.22 |
| Weeks 34–37.6 | 1.68 ± 0.19 | 1.62 ± 0.20 | -0.057 | -0.131 | 0.018 | 0.05 |
| Across gestation | 1.64 ± 0.17 | 1.62 ± 0.17 | -0.014 | -0.054 | 0.027 | 0.51 |

Carotid-femoral pulse wave velocity (cfPWV), augmentation index standardized to heart rate (AIx75), augmentation pressure, time to wave reflection (T1R), subendocardial viability ratio, peripheral systolic blood pressure, peripheral diastolic blood pressure, central systolic blood pressure, central diastolic blood pressure, mean arterial pressure, and pulse pressure amplification (expressed as a ratio) are reported for participants with sub-clinical baseline anxiety symptoms and baseline anxiety symptoms. Data are presented as mean ± standard deviation, unstandardized regression coefficient (B), and 95% confidence intervals (CI), using linear regression for analyses at individual timepoints, mixed-effects models for multiple timepoints across gestation, unadjusted for confounders. Sample sizes per visit: n = 156 (10–13.6 weeks), 135 (14–17.6 weeks), 123 (18–21.6 weeks), 124 (22–25.6 weeks), 126 (26–29.6 weeks), 129 (30–33.6 weeks), and 129 (34–37.6 weeks). P-values were adjusted using Benjamini-Hochberg correction for multiple comparisons across timepoints and the Benjamini-Yekutieli correction for comparisons across mixed-effects models. *Significant at an p-adjusted value <0.05, rounded to nearest hundredth.

**Supplemental Table 5: Odds ratios for preeclampsia given presence of anxiety symptoms in pregnancy**

| **Characteristic** | **OR (95% CI)** | **P value** |
| --- | --- | --- |
| **Anxiety symptoms at baseline** *unadjusted:* | 2.77 (0.84 – 9.18) | 0.09 |
| **Anxiety symptoms at baseline** *adjusted for:* |  |  |
| BMI | 2.47 (0.73 – 8.35) | 0.16 |
| Age | 2.60 (0.77 – 8.75) | 0.12 |
| Race | 2.72 (0.82 – 9.03) | 0.10 |
| Chronic hypertension | 2.68 (0.77 – 9.34) | 0.12 |
| History of preeclampsia | 2.66 (0.79 – 8.91) | 0.11 |
| Family history of preeclampsia | 2.01 (0.56 – 7.27) | 0.29 |
| Diabetes | 2.91 (0.86 – 9.82) | 0.09 |
| **Anxiety symptoms at either timepoint** *unadjusted:* | 3.04 (0.79 – 11.68) | 0.11 |
| **Anxiety symptoms at either timepoint** *adjusted for:* |  |  |
| BMI | 2.79 (0.72 – 10.84) | 0.14 |
| Age | 2.89 (0.75 – 11.18) | 0.13 |
| Race | 2.95 (0.77 – 11.37) | 0.12 |
| Chronic hypertension | 2.92 (0.74 – 11.49) | 0.12 |
| History of preeclampsia | 2.97 (0.77 – 11.45) | 0.11 |
| Family history of preeclampsia | 1.98 (0.47 – 8.31) | 0.35 |
| Diabetes | 3.27 (0.83 – 12.84) | 0.09 |
| **Self-reported history of emotional disorders** *unadjusted:* | 3.91 (1.14 – 13.40) | **0.03*** |
| **Self-reported history of emotional disorders** *adjusted for:* |  |  |
| BMI | 3.86 (1.10 – 13.50) | **0.03*** |
| Age | 3.87 (1.12 – 13.32) | **0.03*** |
| Race | 3.99 (1.16 – 13.75) | **0.03*** |
| Chronic hypertension | 3.83 (1.11 – 13.17) | **0.03*** |
| History of preeclampsia | 3.41 (0.90 – 13.00) | 0.07 |
| Family history of preeclampsia | 3.96 (1.15 – 13.64) | **0.03*** |
| Diabetes | 5.04 (1.35 –18.71) | **0.02*** |

Data presented as odds ratio (OR) and 95% confidence interval (CI). * Significant at p-value <0.05
